# Association Between Gray Matter Microstructure and Glucose Metabolism in Older Adults With Cognitive Decline: A Multimodal Imaging Study

**DOI:** 10.1002/hbm.70661

**Published:** 2026-10-27

**Authors:** Antonietta Canna, Alessandro Pasquale De Rosa, Emanuele Nicolai, Elena Salvatore, Carlo Cavaliere, Angelica Di Cecca, Miriana Migliaccio, Vincenzo Alfano, Sabina Pappatà, Marco Aiello, Alzheimer's Disease Neuroimaging Initiative

**Affiliations:** ^1^ IRCCS SYNLAB SDN Naples Italy; ^2^ Department of Advanced Medical Science University of Naples Federico II Naples Italy

**Keywords:** cognitive impairment, diffusion MRI, glucose metabolism, NODDI

## Abstract

Cortical glucose hypometabolism is a well‐established early marker of neurodegeneration in dementia, mainly reflecting neuronal and synaptic dysfunction. Its relationship with underlying microstructural alterations remains unclear. Here, we combined multi‐shell diffusion microstructural and [^18^F]‐FDG PET data to investigate the possible association between regional cerebral glucose metabolism and microstructural integrity in patients with cognitive decline. Subjects from two independent datasets, an in‐house dataset of 65 cognitively impaired patients from a memory‐clinic cohort and a cognitively impaired cohort retrieved from the ADNI database (advanced ADNI3 protocol), underwent [^18^F]‐FDG PET and MRI scanning. Multiple linear regression models were used to evaluate regional associations between glucose metabolism and gray matter microstructure, as measured by NODDI‐derived metrics, namely neurite density index (NDI) and orientation dispersion index (ODI). Global spatial associations between metabolic and microstructural‐derived maps were investigated via spin tests. Regional analysis showed widespread significant positive associations between glucose consumption and ODI across the cortex and subcortex. In both datasets, the global spatial association between NODDI and metabolism showed the ODI being significantly correlated with glucose metabolism (*ρ* = 0.36, *p*_spin_ < 0.001 for the in‐house dataset; *ρ* = 0.54, *p*_spin_ < 0.001 for the ADNI dataset). Our findings provide new evidence that regional glucose metabolism in cognitive decline is positively associated with alterations in microstructural organization, as reflected by decreased neurite complexity. The significant association suggests that microstructural alterations may parallel the metabolic deficits characteristic of neurodegenerative diseases, pointing to a link between microstructural properties and neuronal metabolic dysfunction.

## Key Points

- In cognitive impairment, a strong association between neurite orientation dispersion (ODI) and glucose metabolism suggests a close link between microstructural complexity and its underlying metabolic processes.
- FDG‐PET metabolism shows robust, widespread positive associations with ODI across cortical and subcortical regions, independently from atrophy.
- The coupling between glucose metabolism and microstructural organization (ODI) is significantly consistent across two independent datasets.

## Graphical abstract

We used multimodal PET‐MRI data to study the gray matter glucose metabolism‐microstructural architecture relationship in cognitively impaired brains. Results showed a significant positive correlation between glucose metabolism and NODDI metrics, particularly with the orientation dispersion index (ODI), in two independent datasets.

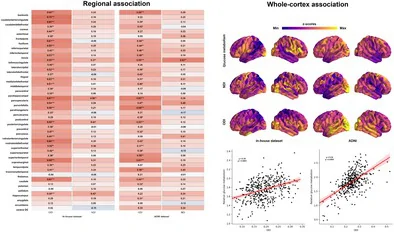

## 1 Introduction

Dementia refers to a progressive cognitive deterioration caused by underlying neuropathological processes that cause widespread neuronal loss (Goel et al. 2022), synaptic dysfunction (Chen et al. 2019), and network disintegration (Pievani et al. 2011) in the brain. It encompasses multiple etiologies, including neurodegenerative, vascular, traumatic, infectious, and metabolic causes. The most common forms are Alzheimer's disease (AD), vascular dementia, Lewy body dementia, and frontotemporal dementia (Gale et al. 2018). At the molecular level, neurodegenerative dementias are often characterized by abnormal protein aggregation (Chen et al. 2021; Wu et al. 2024), for example, amyloid‐β plaques and tau neurofibrillary tangles in AD, α‐synuclein inclusions in Lewy body dementia, or TDP‐43 inclusions in frontotemporal dementia, neuroinflammation, and neurovascular dysfunction, which collectively impair neuronal signaling and cerebral homeostasis (Wang et al. 2023).

A growing body of evidence indicates that altered glucose metabolism is an early marker of dementia. Impairments in neuronal glucose utilization are thought to precede neurodegeneration and may serve as an early and sensitive imaging biomarker for the pathology detection and characterization (Kirvalidze et al. 2022). Under normal physiological conditions, the brain exhibits a disproportionately high rate of glucose consumption relative to its mass, as glucose represents its principal energy substrate (Ishibashi et al. 2016). This metabolic demand supports neuronal function by sustaining synaptic transmission, maintaining ion gradients, and enabling neurotransmitter synthesis (Li and Sheng 2022; Mergenthaler et al. 2013). Consequently, any perturbation in the cerebral metabolic rate of glucose can influence synaptic excitability and neuronal activity (Dienel 2019; Mosconi et al. 2008). In AD, for instance, these metabolic abnormalities emerge early during disease progression; in fact, reductions in glucose utilization have been consistently reported in the hippocampus and posterior cingulate cortex during the prodromal and early symptomatic stages (Chen et al. 2021; Protas et al. 2013). As the pathology advances, the hypometabolic pattern extends to temporo‐parietal association areas and, subsequently, the frontal and occipital cortices (Ferrari et al. 2019). This progressive decline in glucose metabolism closely parallels the loss of synaptic density and function (van Aalst et al. 2021), highlighting the tight coupling between energy metabolism and the underlying neuronal substrate, that is, the specific networks of neurons, dendrites, and synapses that support brain function and cognition. This neuronal substrate can be characterized both at the macrostructural level, where volumetric atrophy serves as a late marker of neuronal loss, and at the microstructural level, which captures neurite density (dendrites and axons) and organization before atrophy becomes evident (Vogt et al. 2020). Microstructural alterations, such as reduced neurite density or changes in orientation dispersion, may parallel neuronal degeneration and reflect early network remodeling, providing a sensitive index of synaptic and functional health. However, the microstructural correlates of these metabolic abnormalities remain unknown (Strom et al. 2022). Clarifying the relationship between cerebral hypometabolism and changes in neuronal architecture, which may precede detectable atrophy in cortical and deep gray matter (DGM), is essential for understanding the mechanisms underlying cognitive decline.

Recent advances in diffusion MRI have enabled the in vivo characterization of brain microstructure with unseen specificity. Among these techniques, neurite orientation dispersion and density imaging (NODDI) has emerged as a cutting‐edge approach (Zhang et al. 2012) capable of disentangling different microstructural components of the neuronal substrate. Unlike conventional diffusion tensor imaging (DTI), which provides nonspecific indices of diffusivity and is more suitable for characterizing white matter properties, NODDI models the diffusion signal to estimate neuroanatomical microstructure characteristics, such as neurite density and orientation dispersion. These metrics provide a means to quantify the axonal and dendritic complexity of gray matter (GM) tissue, serving as valuable proxies for neuronal and synaptic architecture (Zhang et al. 2012). In dementia, NODDI can be used for detecting early microstructural alterations that accompany and contribute to glucose hypometabolism (Gallagher et al. 2023; Yu et al. 2024). Linking metabolic markers associated with neurodegeneration to microstructural features of cortical and subcortical GM may provide novel insights into the relationships between energy metabolism and neuritic integrity.

In this study, we combined diffusion‐based microstructural imaging and [^18^F]‐fluorodeoxyglucose (FDG) PET data in two independent datasets, an in‐house cohort of memory‐clinic patients and a cognitively impaired cohort from the Alzheimer's Disease Neuroimaging Initiative's (ADNI3). In addition, since resting‐state functional networks reflect baseline neuronal activity in the absence of task‐directed behavior or external stimulation and glucose metabolism serves as a proxy for neuronal activity, we also explored its relationship with the underlying microstructural properties of canonical resting‐state networks obtained by using a functional parcellation (Yeo et al. 2011).

## 2 Materials and Methods

### 2.1 Participants

A total of 65 participants were retrospectively selected from a cohort of memory‐clinic patients with different levels of cognitive impairment varying from subjective cognitive decline (SCD) to objective (mild cognitive impairment [MCI], AD, and non‐AD dementia) forms. All participants underwent neuroimaging and neuropsychological evaluation at the Istituto di Ricovero e Cura a Carattere Scientifico (IRCCS) SYNLAB SDN (Naples, Italy). Inclusion criteria for this study were the presence of at least the [^18^F]‐FDG PET, structural, and multishell diffusion MRI scans, and the absence of other neurologic diseases or brain focal lesions (e.g., strokes, tumors, and trauma), claustrophobia, and MRI contraindications (e.g., metallic implants, pacemakers). Study approval was obtained from the local ethics committee. All participants provided written informed consent in accordance with the Declaration of Helsinki.

A second validation dataset was obtained from the ADNI database (adni.loni.usc.edu). All participants with cognitive impairment from the ADNI3 protocol were considered for the inclusion of both multi‐shell diffusion and [^18^F]‐FDG PET acquisition, giving us a total of 76 initial participants. After quality control to assess scan completeness, motion and susceptibility artifacts, mis‐registrations, and missing b‐shells, 67 participants were retained for the analysis. Demographic and clinical characteristics of the two datasets are summarized in Table 1.

**TABLE 1:** Demographic information of the two cohorts.

	In‐house dataset	ADNI3
	*N* = 65	*N* = 67
Age (years)	67 ± 7.88	73 ± 7.24
Gender (female)	30	26
MMSE	23.4 ± 5.55	27.1 ± 3.10
Diagnosis		
MCI	26	59
AD	22	8
SCD	6	—
Non‐AD dementia	11	—
Amyloid status (positivity)	34	37

### 2.2 PET Acquisition

For the in‐house dataset, PET data were acquired with a GE Discovery 710 PET‐CT system. Static 20‐min brain images were acquired in 3D iterative ordered‐subset expectation maximization (OSEM) using attenuation‐weighted iterative reconstruction (7 iterations, 36 subsets), 30 min after the injection of about 2.6 ± 0.1 MBq/Kg [^18^F]‐FDG. Image volume consisted of 47 slices with an in‐plane resolution of 1.2 mm and a slice thickness of 3.3 mm. Emission data were corrected for randoms, dead time, and scatter, while attenuation correction was performed using the CT scan. For the ADNI3 dataset, the PET protocol specifics are available at (https://adni.loni.usc.edu/data‐samples/adni‐data/neuroimaging/pet).

### 2.3 MRI Acquisition

For the in‐house dataset, MRI was performed using a 3T Philips Achieva dStream system (Philips Healthcare, Best, The Netherlands) (*N* = 30) or a 3T PET/MR Siemens Biograph mMR system (Siemens Healthcare, Erlangen, Germany) (*N* = 35) with a harmonized protocol, which included: a 3D sagittal T1‐weighted (T1‐w) sequence; a spin echo echo‐planar imaging sequence for diffusion‐weighted imaging (DWI). Multishell diffusion data were acquired in both posterior to anterior (A ≫ P) and anterior to posterior (P ≫ A) phase‐encoding directions. P ≫ A data included two shells (*b* = 1000 s/mm^2^ and *b* = 2000 s/mm^2^) with 30 diffusion encoding directions each, in addition to 7 non‐DWI (*b* = 0 s/mm^2^) acquired every 11 volumes. A ≫ P data included 3 non‐DWIs. Participants from the ADNI3 dataset were scanned with the ADNI 3.0 Advanced MRI scanning protocol, the first ADNI protocol to include multi‐shell acquisition. Imaging protocol included a magnetization prepared rapid acquisition gradient echo (MPRAGE) sequence and an echo‐planar imaging sequence for DWI MRI. For multishell diffusion, P ≫ A data included three shells (6 directions at *b* = 500 s/mm^2^, 48 directions at *b* = 1000 s/mm^2^, and 60 directions at *b* = 2000 s/mm^2^), in addition to 13 non‐DWI (*b* = 0 s/mm^2^) volumes. For both datasets, DWI was acquired with an adaptive coil combination in the Siemens based protocol. Image acquisition parameters for both datasets are summarized in Table 2.

**TABLE 2:** MRI acquisition parameters.

	In‐house dataset (Siemens)	In‐house dataset (Philips)	ADNI
*N*	35	30	67

T1‐w			
Sequence	Turbo fast low‐angle‐shot	Turbo field echo	MPRAGE
Spatial resolution (mm^3^)	1 × 1 × 1	1 × 1 × 1	1 × 1 × 1
Number of slices	176	180	256
TR (ms)	2300	8.1	2300
TE (ms)	3	3.7	2.98
TI (ms)	900	NA	900

DWI			
Spatial resolution (mm^3^)	2.5 × 2.5 × 2.5	2.5 × 2.5 × 2.5	2 × 2 × 2
Number of slices	60	60	81
TR (ms)	9200	8400	3400
TE (ms)	96	85	71

### 2.4 Image Processing

Dynamic [^18^F]‐FDG PET frames from ADNI3 participants were motion corrected and summed into a single static image. All static PET images were then rigidly coregistered to the corresponding T1‐w image and spatially normalized to the common Montreal Neurological Institute (MNI) template space. Maps of relative glucose metabolism for each participant were obtained by voxel‐wise normalization using the pons as reference region (Allocca et al. 2021).

T1‐w images were preprocessed with the standard FreeSurfer pipeline (8.1.0 release) for cortical reconstruction and volumetric segmentation (Fischl 2012). Diffusion data were preprocessed with MRtrix3 (Tournier et al. 2019). Preprocessing included denoising, susceptibility distortion correction with reversed phase‐encoding, eddy current motion correction, bias field correction, and Rician noise correction (Andersson et al. 2003; Andersson and Sotiropoulos 2016; Koay et al. 2009; Smith et al. 2004; Veraart et al. 2016). We fitted the NODDI model to derive brain microstructural maps representing the volume fraction of the intracellular compartment (neurite density index [NDI]) and the angle variation and configuration of the neurite structure (orientation dispersion index [ODI]). The NODDI model was fitted with the accelerated microstructure imaging via convex optimization (AMICO) toolbox (Daducci et al. 2015) and the tissue‐weighted mean method (Parker et al. 2021). Since the default parallel diffusivity of the NODDI model (i.e., *d*_||_ = 1.7 × 10–3 mm^2^/s) is suboptimal in GM, as demonstrated by Guerrero et al. (2016, 2019), we used the optimized parallel diffusivity value of *d*_||_ = 1.1 × 10–3 mm^2^/s resulted from a large study from Fukutomi et al. (Fukutomi et al. 2018) on HCP data (505 healthy subjects). The tissue‐weighted mean reduces estimation bias in regional NODDI metrics by weighting each voxel by its tissue fraction, thereby accounting for CSF partial volume contamination that would otherwise bias conventional voxel‐equally weighted ROI means, particularly, in periventricular regions and in subjects with brain atrophy.

We performed two types of analyses: the primary participant‐level analysis at regional level and the group‐level spatial correspondence analysis across the whole cortex. For the participant‐level analysis, PET and NODDI maps (using the B0 image for the latter) were co‐registered to the corresponding T1w image with linear interpolation. All co‐registrations were visually checked for quality control. Median values of relative glucose metabolism and NODDI metrics were extracted from the automatically parcellated Desikan–Killiany atlas (consisting of 68 bilateral cortical regions and 16 DGM structures) for the regional based analysis and from the 400‐parcels Schaefer atlas in the T1‐w native space for the network‐based analysis (see Supplementary Materials). For the group‐level analysis, each image was nonlinearly registered to the MNI152 standard space. All normalizations were visually checked for quality control. In MNI space, we first regressed out the effects of age, gender, and scanner from each voxel of the spatially normalized PET and NODDI images. Median values were then extracted from each region of the 400‐parcels Schaefer atlas across all participants to obtain parcellated brain maps for relative glucose metabolism, NDI, and ODI (Schaefer et al. 2018).

### 2.5 Statistical Analysis

Multiple linear regression models were used to investigate the regional relationship between relative glucose metabolism and microstructural properties, while controlling for age, gender, and scanner effects. Additionally, regional volumes (normalized by intracranial volume) were included into the linear models as nuisance covariates to regress out the effect of regional atrophy and to mitigate potential confounding effect of partial volume. Results were considered as statistically significant for *p* < 0.05, after false discovery rate (FDR) correction to account for multiple comparisons. FDR correction was applied across all regions separately for ODI‐ and NDI‐related analyses. For the in‐house dataset (which is more clinically heterogeneous than the ADNI3), we tested whether the association between PET and NODDI metrics depended on the diagnosis by fitting additional linear models including an interaction term between NDI/ODI and diagnosis, while adjusting for age, gender, regional volume and scanner. *Z*‐score normalization was applied to the median values before the statistical analyses. To evaluate the consistency of regional associations between the relative gluose metabolism and NODDI metrics in the two independent datasets, cross‐cohort agreement was assessed separately for ODI and NDI by calculating Spearman rank correlations (*ρ*) between the ROI‐wise standardized beta estimates, with 95% bootstrap confidence intervals. More in detail, for each cortical and subcortical ROI of the Desikan–Killiany atlas, we extracted the standardized beta coefficients from cohort‐specific linear models adjusted for age, gender, scanner, and regional volume. Bootstrap resampling (5000 iterations) was used to derive 95% confidence intervals around both correlation metrics. Last, the proportion of ROIs showing concordant direction of effect (i.e., same sign of beta) across cohorts was also calculated.

For the group‐level spatial correspondence analysis, we used spatial similarity analysis, separately for each independent dataset, to investigate whether there was a significant association between relative glucose metabolism and cortical microstructure. Statistical significance of spatial similarity across cortical brain regions was assessed with spatial autocorrelation‐preserving permutation tests, known as spin tests (Váša and Mišić 2022) applied on the age, gender, and scanner adjuster median values extracted from each region of the 400‐parcels Schaefer atlas in the MNI space. Using *neuromaps* (Markello et al. 2022), spherical projection of the *fsLR* surface defined spatial coordinates for each parcel by selecting the coordinates of the vertex closest to the centroid for each parcel. These parcel coordinates were then randomly rotated and original parcels reassigned to the value of the closest rotated parcel for *N* = 1000 repetitions. For parcels where the medial wall is the closest, the value of the next closest parcel is assigned instead. Following these steps, we obtained a series of randomized brain maps that had the same values and spatial autocorrelation as the original map, while the relationship between values and their spatial location were permuted. These maps were then used to generate null distributions of Spearman's *ρ* correlation coefficients for assessing statistical significance of spatial correlation. Similarly to other works, we performed this analysis at the parcel resolution rather than the vertex resolution to avoid upsampling the data and to each hemisphere separately (Hansen et al. 2022). These surface‐based permutations were then applied to the corresponding volumetric parcel values (i.e., the same Schaefer‐400 parcels in MNI volume space) to compute empirical *p*‐values. Thus, the spin test provides a valid null model for the spatial correlation between two volumetric maps, without requiring the maps themselves to be projected to the surface. Since the spin‐test procedure implemented in the neuromap tool operates on the parcellated region‐wise values in the MNI space (Hansen et al. 2022), we did not apply any volume‐to‐surface transformation for the group‐level statistical analysis.

## 3 Results

### 3.1 Significant Glucose Metabolism–NODDI Associations Across Cortical and DGM Regions

Participant‐level regional analysis performed within the anatomical Desikan–Killiany parcels showed widespread significant associations between relative glucose metabolism and NODDI parameters both considering the cortical and subcortical regions as shown in Figure 1. Regression coefficients between ODI and glucose metabolism were significant in nearly all cortical structures, while associations between NDI and glucose metabolism reached significance in only a limited number of regions across the two datasets (Figure 1). No significant interactions between NDI or ODI and diagnosis were observed in predicting glucose metabolism. Last, widespread significant associations were observed in the functional networks considering the correlations between relative glucose metabolisms and NODDI‐derived metrics in both datasets. Details for this analysis are provided in the Supplementary Materials.

**FIGURE 1:**
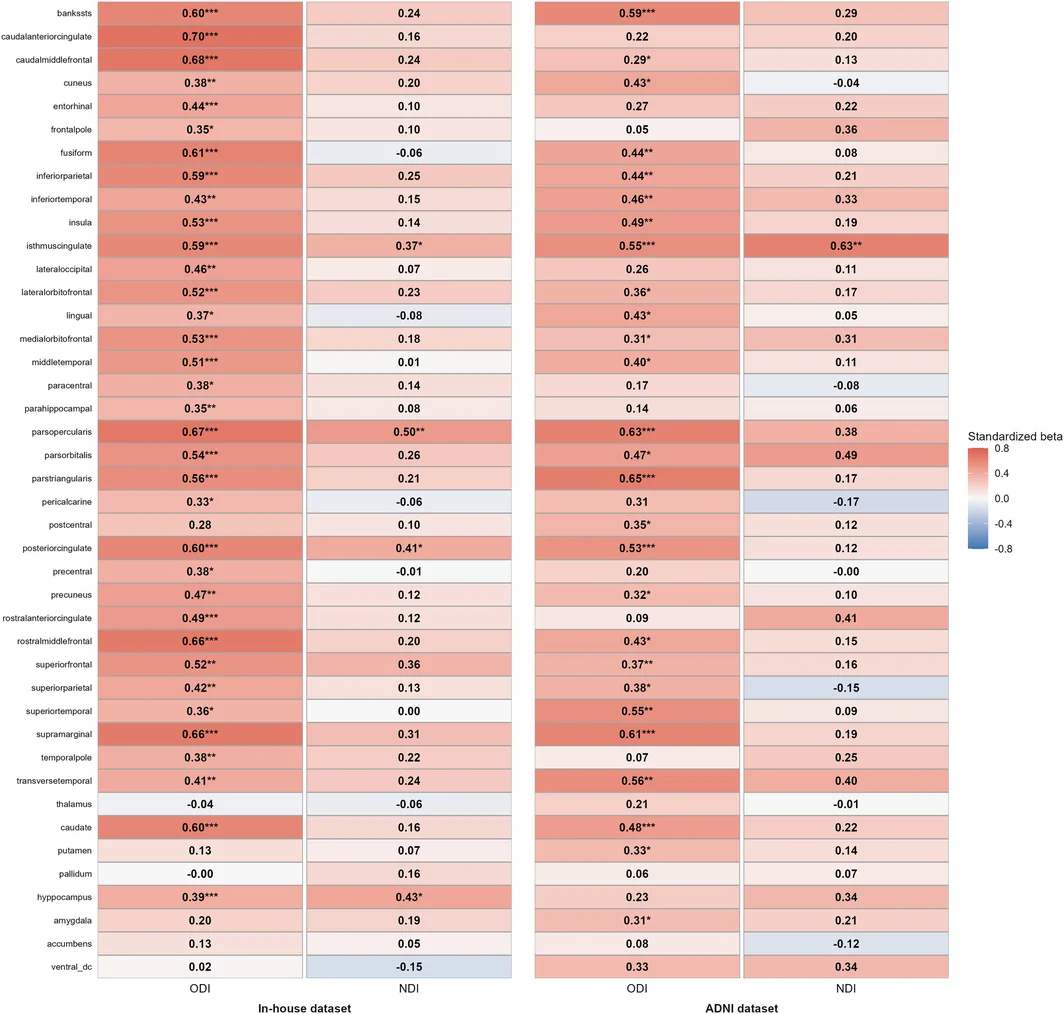
Regression coefficients between relative glucose metabolism and NODDI metrics for regions from the Desikan–Killiany parcellation. Values in the table represent the standardized beta values from the linear regression models. *p < 0.05, **p < 0.01, ***p < 0.001.

### 3.2 Cross‐Cohort Agreement of Regional Effect Estimates

The cross‐cohort agreement for the ODI association with relative glucose metabolism showed statistically significant agreement in regional effect magnitude between cohorts. The Spearman correlation was *ρ* = 0.54, with a 95% bootstrap confidence interval of [0.27, 0.74] and *p* < 0.001. The direction of the association was consistent in 40 out of 42 regions corresponding to 95.2% directional agreement. The cross‐cohort analysis agreement for the NDI association with glucose metabolism also showed significant weaker agreement, in regional effect magnitude with a Spearman correlation of *ρ* = 0.53, a 95% bootstrap confidence interval of [0.24, 0.73] and *p* < 0.001. In this case, 35 out of 42 regions showed the same effect direction, corresponding to 83.3% directional agreement. A graphical overview of these results is shown in Figure 2. Each ROI has been classified into one of four significance patterns based on FDR‐corrected *p*‐values within each cohort (*p*_FDR_ < 0.05): (1) FDR‐significant in both cohorts, (2) FDR‐significant only in the in‐house cohort, (3) FDR‐significant only in ADNI, or (4) not FDR‐significant in either cohort.

**FIGURE 2:**
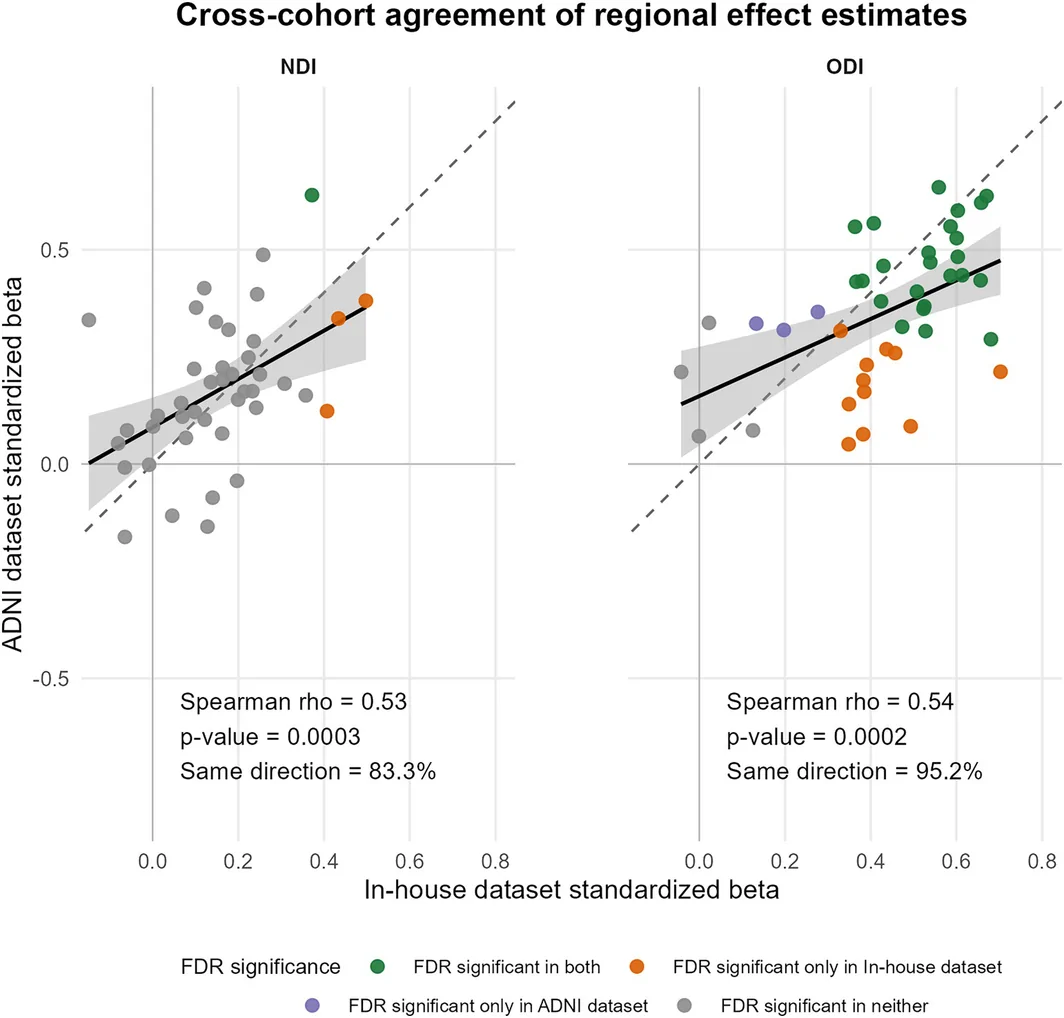
Scatterplots reporting the standardized betas relative to the in‐house dataset (*x*‐axis) and to the ADNI dataset (*y*‐axis), for the ODI (left) and the NDI (right). Each point represents one region from the Desikan–Killiany parcellation. Plots include a line of equality, a linear regression fit with 95% confidence band, and annotations reporting Spearman *ρ*, associated *p*‐value, and percentage of ROIs with concordant direction of effect. Points are color‐coded according to the four significance patterns described in Figure 1.

### 3.3 Glucose Metabolism—ODI Spatial Association Across the Whole Cortex

Figure 3 reports the z‐scores of relative glucose metabolism and NODDI‐derived metrics in the 400‐parcels Schaefer atlas projected on the *fsaverage* surface. Globally, we found a significant spatial correlation between relative glucose metabolism and ODI in both datasets (*ρ* = 0.36, *p*_spin_ < 0.0001 for the in‐house dataset; *ρ* = 0.54, *p*_spin_ < 0.0001 for the ADNI dataset; Figure 4). No significant association was found with NDI (*ρ* = −0.02, *p*_spin_ > 0.05 for the in‐house dataset; *ρ* = −0.10, *p*_spin_ > 0.05 for the ADNI dataset; Figure 4).

**FIGURE 3:**
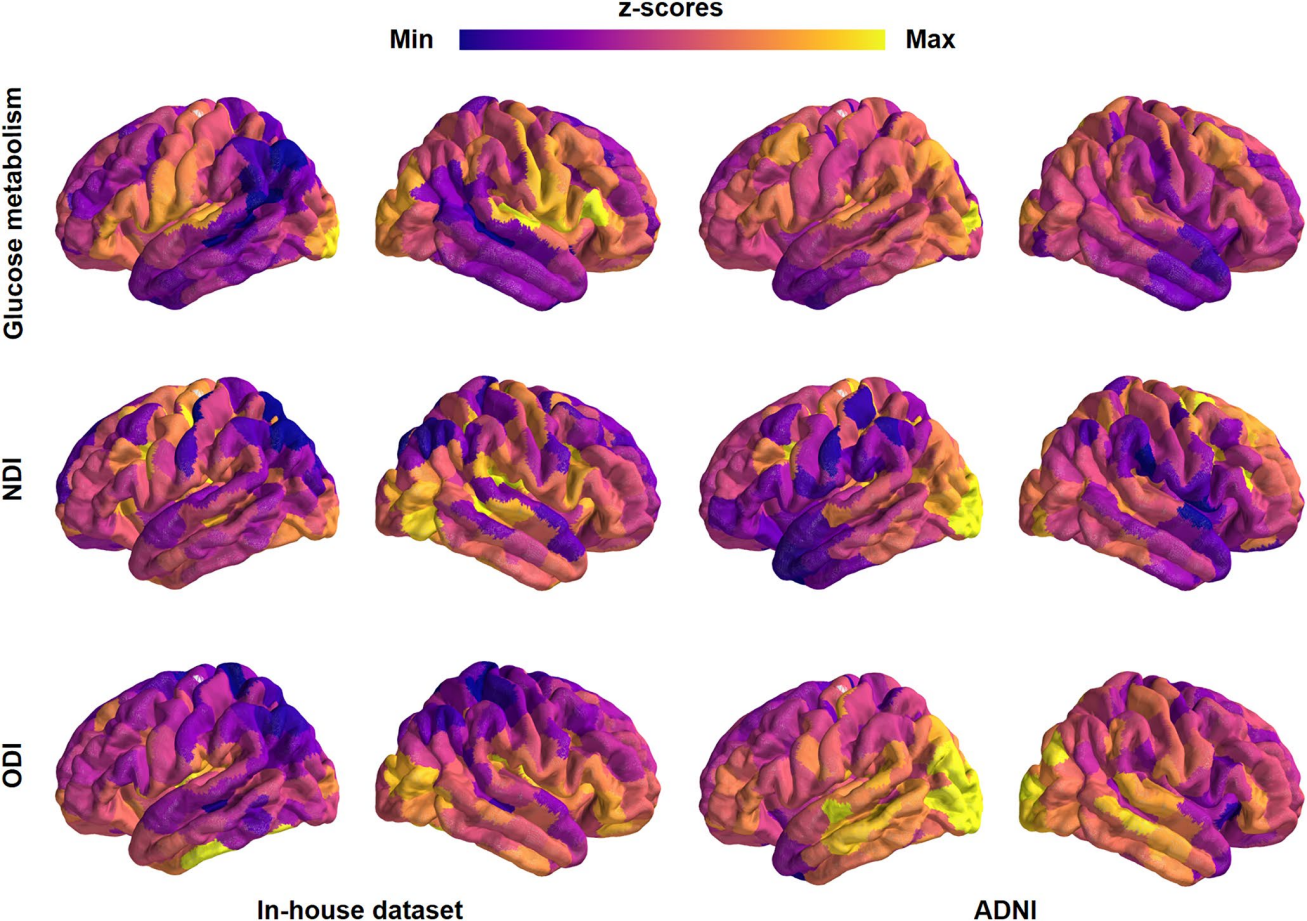
Brain maps for PET and NODDI‐derived metrics. Parcellated maps for relative glucose metabolism, NDI, and ODI from the in‐house dataset (left panel) and the ADNI dataset (right panel). The same colorbar scale was applied for all brain maps.

**FIGURE 4:**
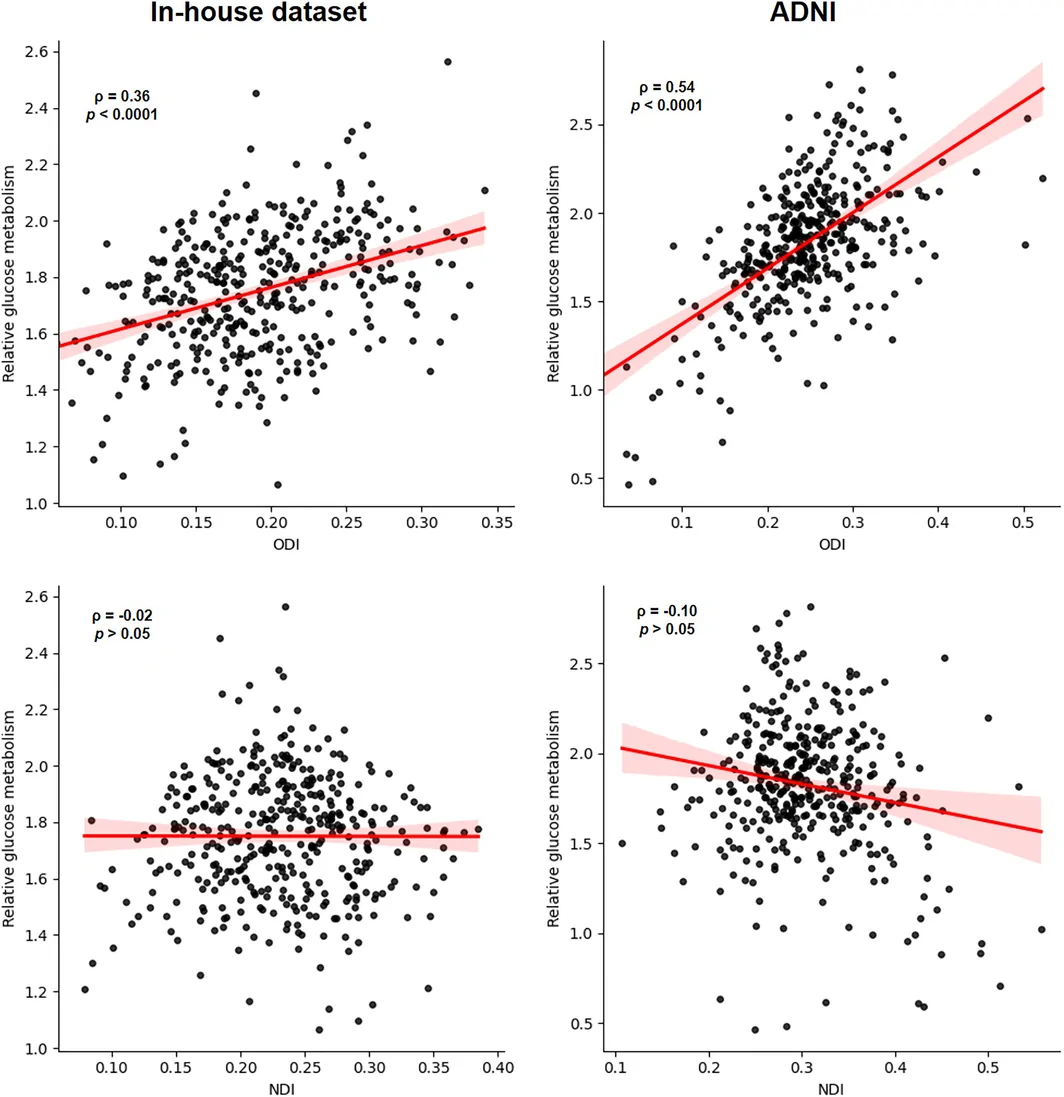
Scatter plots between PET and NODDI‐derived metrics. Scatter plots across the 400 parcels of the Schaefer atlas between relative glucose metabolism (*y*‐axis) and NODDI‐derived metrics (*x*‐axis) for the in‐house dataset (left panel) and the ADNI dataset (right panel).

## 4 Discussion

In this study, we combined multishell diffusion MRI and [^18^F]‐FDG PET imaging to investigate the relationship between glucose metabolism and microstructural markers in cortical and subcortical GM regions of older adults with cognitive decline from two independent datasets. Given the fundamental role of glucose consumption in neuronal activity and function, we conducted statistical analyses at two spatial scales: regionally with the anatomical and participant‐specific Desikan–Killiany atlas and globally across the whole cortex.

In both datasets, ODI showed widespread regional associations with glucose metabolism, whereas NDI correlations reached significance in a limited number of regions. Our results also revealed a significant global positive correlation between glucose metabolism and cortical microstructure complexity, as measured by ODI (*ρ* = 0.36, *p*_spin_ < 0.001 for the in‐house dataset; *ρ* = 0.54, *p*_spin_ < 0.001 for the ADNI dataset). At a functional network level, the strongest positive associations with ODI emerged within transmodal functional networks. Considering a more quantitative measurement of the cross‐cohort agreement, significant results were obtained for both NODDI metrics, suggesting that our findings are significantly consistent across the two datasets. It is important to highlight that significant consistencies across datasets were found in both ODI‐ and NDI‐related analyses as revealed by the cross‐cohort agreement of regional effect estimates. However, for ODI, the cross‐cohort concordance was accompanied by widespread significant regional associations between relative glucose metabolism and ODI in both datasets. For NDI, in contrast, the significant cross‐cohort agreement indicates that the pattern of ROI‐wise standardized beta estimates was consistent between the two datasets, even though the NDI association with relative glucose metabolism was not statistically significant in almost any individual region.

Importantly, all regional results were obtained after adjusting for local atrophy as measured by regional volumes with T1w images, suggesting that the relationship between glucose metabolism and neurite complexity may not be exclusively driven by neuronal or synaptic loss. However, this result should be interpreted with caution, since DWI voxels had dimensions comparable to cortical thickness (i.e., 2/2.5 mm), and we cannot exclude partial volume contamination from low‐ODI WM.

One possible explanation about the significant FDG‐ODI association may be a dendritic reorganization possibly associated with metabolic dysfunction and/or neurodegeneration in patients with different degrees of cognitive impairment. The positive association between ODI and glucose metabolism may reflect multiple, partially overlapping biological mechanisms. Specifically, lower ODI reflects greater neurite orientation coherence and in conjunction with hypometabolism may be compatible with microstructural remodeling associated with aging or neurodegeneration (Nazeri et al. 2015). However, ODI alone cannot determine whether this pattern reflects preserved neuritic organization, selective changes in differently oriented neuritic processes, or broader alterations in neuritic and dendritic architecture. Conversely, increased ODI may indicate structural reorganization or neuritic sprouting, potentially associated with compensatory mechanisms (Niu et al. 2025). Inflammatory processes could also contribute, as reactive gliosis and microglial proliferation increase the number of cellular processes oriented in multiple directions, thereby increasing angular dispersion (Plank et al. 2025). Although glucose metabolism is primarily interpreted as a marker of neuronal and synaptic activity, a proportion of the signal may arise from glial metabolism. Thus, particularly, at specific disease stages, neuroinflammatory processes may also contribute to the observed associations (Sacco et al. 2020). In addition, the co‐occurrence of hypometabolism and synaptic dysfunctions may result from a shared pathological driver, and thus producing a significant positive association.

Overall, while these interpretations remain speculative and cannot be confirmed using the current cross‐sectional imaging data, our findings suggest that ODI is globally associated with glucose metabolism and may capture microstructural changes linked to neurodegeneration, dysfunction, compensatory remodeling, and possibly neuroinflammation. Future research will be needed to validate these results using longitudinal designs, neuropathological data, or additional molecular and cellular biomarkers. In addition, at the regional level, ODI was more widely correlated to FDG than NDI. The lack of significant correlations between NDI and FDG could possibly indicate that ODI, known as a marker of neurite organization, may capture much more information (both structural and functional) than NDI, that is, by definition, a marker of structural density. However, ODI could also be more sensitive to partial volume effects and therefore be additionally dependent on tissue atrophy through WM contamination.

FDG‐PET is a well‐established in vivo marker of cerebral glucose metabolism, closely related to synaptic activity and neuronal integrity. Disease‐specific patterns of hypometabolism characterize AD and other neurodegenerative dementias (Minoshima et al. 2022), and reductions in FDG uptake are commonly interpreted as a proxy of neurodegeneration and synaptic dysfunction (Minoshima et al. 2022). In addition, evidence suggests that FDG‐PET signals also reflect, at least in part, glucose consumption in astrocytes (Zimmer et al. 2017) and microglia (Xiang et al. 2021). Interestingly, it has been recently proposed that AD can be described as a disorder of metabolic inflexibility that leads to bioenergetic failure (Constantino et al. 2025). In the healthy brain, metabolic flexibility allows neurons, glia, and vascular cells dynamically share and switch between different fuel sources, such as glucose, lactate, and fatty acids, to meet functional demands. However, this metabolic adaptability is progressively lost in pathological conditions, such as AD, as cellular metabolism is reprogrammed to support neuroinflammatory and disease‐associated processes at the expense of neuronal function (Constantino et al. 2025). Microglia, in particular, upregulate glycolytic metabolism and prioritize immune functions, which actively deplete the brain's energy supply (Ghosh et al. 2018; Nirakis et al. 2025). These adaptations are initially seen as a compensatory mechanism but ultimately lead the brain to a rigid metabolic program that deprioritizes neuronal support (Chausse et al. 2024). Thus, an initial phase of hypermetabolism is associated with inflammation, but, as neurodegeneration progresses, this metabolic activity declines, ultimately leading to neuronal dysfunction (Constantino et al. 2025). Critically, these changes promote amyloid‐β production, tau release, and protein aggregation, further increasing metabolic rigidity and establishing a feedback loop between hypometabolism and protein aggregation and deposition. Consistent with this hypothesis, previous findings showed a positive correlation between increased FDG uptake and microglial activation (TSPO‐PET) in relatively preserved brain regions of patients with AD and four‐repeat tauopathies, whereas this association was absent in areas with marked FDG hypometabolism, where PET changes likely reflect predominant neuronal dysfunction (Xiang et al. 2021).

FDG‐PET and structural MRI have been introduced as markers of neurodegeneration in the amyloid–tau–neurodegeneration biomarker framework (Jack et al. 2016). However, spatial dissociation between atrophy and hypometabolism suggests that they measure different processes. Functional disconnection from primary atrophic regions may contribute to regional hypometabolism, among other factors (Mutlu et al. 2016). Results from a recent meta‐analysis of 4218 MCI/AD patients (Dang et al. 2025) showed minimal spatial overlap pattern between hypometabolism and atrophy, further supporting the hypothesis that these reflect two distinct aspects of neurodegeneration. Nevertheless, these two processes, not only are spatially distinct but also occur at different stages of disease progression, suggesting that neuronal reshaping may precede neuronal loss. Therefore, we hypothesize that hypometabolism may be associated with a rearrangement of underlying neuronal complexity, rather than being solely a consequence of atrophy.

Our study combining NODDI with PET imaging is validated in two independent datasets and contributes to recent literature that analyzes the relationship between NODDI metrics and the major pathological signs. For example, in the GM of patients with young‐onset AD, decreased NDI and ODI values have been reported in the temporal cortex and precuneus, with lower cortical NDI correlating with greater cognitive impairment (Parker et al. 2018). Notably, Parker et al. (2018) also described reduced NDI in the precentral gyrus, a region typically preserved from overt macroscopic atrophy but nevertheless susceptible to AD pathology and linked to clinical deficits (Albers et al. 2015). The recent post‐mortem study of Alasmar et al. (2026) reported an association between DTI and markers of amyloid and tau pathology, as well as vascular neuropathology and cognitive status, supporting the potential value of DWI as a noninvasive and more accessible approach to assess the severity of AD neuropathology in conjunction with other emerging biomarkers. In line with this perspective, our finding of a close coupling between ODI and cerebral glucose metabolism further supports the relevance of NODDI metrics. ODI may represent a promising candidate imaging marker that could complement FDG‐PET in the evaluation of cognitive impairment.

Importantly, these relationships between relative glucose metabolism and NODDI metrics were investigated at different spatial scales: at participant‐level using the subject‐specific parcellation of the Desikan–Killiany atlas and at group‐level analysis by using the 400 parcels of the Schaefer atlas. The participant‐level analysis is the more direct inferential analysis because both modalities were acquired in the same individuals, thus allowing us to evaluate more precisely the association between NODDI metrics and glucose metabolism; the group‐level spatial correspondence analysis is more descriptive since it collapses both subjects and cortical regions and allows us to observe spatial distribution across the cortical parcels of neurophysiological parameters (Hansen et al. 2022). However, even though the participant‐level analysis provides a more direct assessment of the association between NODDI metrics and glucose metabolism, we cannot relate it to a specific cellular or pathophysiological mechanism.

To reduce the spatial transformations applied to the PET and NODDI data, the participant‐level analysis has been done in the space of the T1w image by using the FreeSurfer's Desikan–Killiany parcellation, that is subject‐oriented and, thus, takes into account possible cortical atrophy. For the group‐level analysis, we used a surface‐based spin‐test approach (Hansen et al. 2022) that operates on parcellated, region‐wise values. As implemented in the neuromap tool, region labels are permuted on the cortical surface to generate spatially constrained null distributions that preserve the intrinsic spatial autocorrelation of the cortical sheet (Hansen et al. 2022). These surface‐based permutations are then applied to the corresponding volumetric parcel values to compute empirical *p*‐values. Parcellation was performed in MNI volume space using the anatomically‐informed Schaefer‐400 functional atlas. Spin‐test permutations were generated on the fsLR spherical surface using the Schaefer parcel centroids and subsequently applied to the volumetric parcel values to construct the null distribution. This approach preserves the spatial autocorrelation structure of the cortex while avoiding unnecessary volume‐to‐surface interpolation of the raw imaging data.

The current study has some limitations. First, we could not compare our cohorts with a matched control group: for the in‐house dataset, the Italian law discourages PET scanning of healthy individuals, while for the ADNI3 dataset, the small number (*N* = 4) of cognitively normal participants with both multishell DWI and FDG‐PET could not allow for a meaningful statistical comparison. Second, our cohorts included people with different and heterogeneous clinical diagnosis, albeit all with cognitive impairment. However, results in the in‐house were consistent when we added the diagnosis as an interaction term in the linear regression models, suggesting that the relation between NODDI metrics and relative glucose metabolism does not change with the clinical diagnosis. However, we want to emphasize that in this study we cannot precisely attribute the spatial variations of glucose metabolism to structural variations in the NODDI metrics (or viceversa) as a function of the specific diagnosis. For such an analysis, higher sample sizes for each diagnosis would be needed and, more critically, a group of healthy participants would be necessary to individuate through specific (regional or voxel‐wise) group‐comparisons possible regions in the brain that are affected in each diagnosis, in terms of glucose metabolism and/or microstructure, and to further explore whether and how the patterns of differences in the NODDI metrics are coupled to corresponding differences in metabolism (or vice versa). Discrepancies in observed global patterns in the two datasets might be due to the different clinical populations and other technical differences such as in the acquisition protocols (TR, TE, and voxel sizes 2 vs. 2.5 mm iso) and in the scanner hardware.

As last consideration, brain microstructure models for GM should also take into account the non‐negligible water exchange in cell bodies (i.e., soma), particularly, at high b‐values, where the standard two‐compartments model may not hold true. While this may be mitigated in the datasets used here (since the maximum *b*‐value is 2000 s/mm^2^), more recent biophysical models, such as the soma and neurite density imaging (SANDI) model, can better describe GM microstructure (Palombo et al. 2020).

In conclusion, this study revealed an association between glucose metabolism and GM microstructure in dementia, particularly, with microstructural complexity measured by ODI. These correlations were independent of local volumes as measured by T1w images, suggesting that metabolic alterations reflect changes in neuronal architecture beyond tissue loss. Our findings therefore support the idea that metabolic dysfunction is associated with microstructural reshaping and may represent a key mechanism in the pathophysiology of neurodegeneration. These results provide new evidence that NODDI‐derived metrics, particularly, ODI, are associated with neuronal glucose metabolism, while also highlighting the potential of combining diffusion MRI and PET to improve the structural and functional characterization of cognitive impairment.

## Funding

This work was carried out within Project No. PNRR‐MCNT2‐2023‐12378268 (CUP I63C24000490001), funded by the European Union – NextGenerationEU and the Italian Ministry of Health under the National Recovery and Resilience Plan (PNRR), Mission 6, Component 2, Investment 2.1: “Rafforzamento e potenziamento della ricerca biomedica del SSN”.

## Supporting information


**Supplementary Table 1.** Significant regional associations between relative glucose metabolism and ODI in the resting‐state functional networks for both datasets.

## Data Availability

The ADNI3 dataset used in this study is publicly available at https://adni.loni.usc.edu. The in‐house dataset is available upon reasonable request to the corresponding author M.A. (marco.aiello@synlab.it).
