# Supplementary material for: Association Between Gray Matter Microstructure and Glucose Metabolism in Older Adults With Cognitive Decline: A Multimodal Imaging Study
Source: Hum Brain Mapp. 2026 Oct 27;47(15):e70661. doi: 10.1002/hbm.70661 (PMC13712218; doi:10.1002/hbm.70661)
Supplement: Supplementary file 1 [file HBM-47-e70661-s001.docx]

**Supplementary Materials**

**Network analysis**

For each participant, the Schaefer atlas was mapped from the *fsaverage* template to the individual's cortical surface, preserving the deterministic parcel labels, and then converted to a volumetric parcellation. In the volume space, median values of the 7 functional networks were extracted and used for the statistical analyses.

***Glucose metabolism–NODDI association in functional networks***

We found a significant association between NDI and glucose metabolism in the left attention network (*ꞵ* = 0.38, *R^2^* = 0.31, *p* < 0.05), left salience network (*ꞵ* = 0.33, *R^2^* = 0.19, *p* < 0.05), left limbic network (*ꞵ* = 0.44, *R^2^* = 0.38, *p* < 0.05), left control network (*ꞵ* = 0.51, *R^2^* = 0.23, *p* < 0.01), left default mode network (*ꞵ* = 0.39, *R^2^* = 0.28, *p* < 0.05), the right visual network (*ꞵ* = 0.48, *R^2^* = 0.35, *p* < 0.01), right attention network (*ꞵ* = 0.35, *R^2^* = 0.21, *p* < 0.05), right salience network (*ꞵ* = 0.33, *R^2^* = 0.16, *p* < 0.05), right control network (*ꞵ =* 0.46, *R^2^* = 0.25 *p* < 0.01) and in the right default mode network (*ꞵ* = 0.42, *R^2^* = 0.27, *p* < 0.05) for the in-house dataset, and in the left visual network (*ꞵ* = 0.36, *R^2^* = 0.28, *p* < 0.05) and in the right visual network for the ADNI dataset (*ꞵ* = 0.43, *R^2^* = 0.40, *p* < 0.01). More widespread significant correlations with ODI were found in both dataset (Supplementary Table 1).

***Supplementary Table 1.*** *Significant regional associations between relative glucose metabolism and ODI in the resting-state functional networks for both datasets.*

| **In-house dataset** | | | |
| --- | --- | --- | --- |
| **Region** | ***ꞵ*** | ***R^2^*** | ***p*** |
| Left Visual Network | 0.43 | 0.23 | <0.01 |
| Left Sensorimotor Network | 0.48 | 0.16 | <0.01 |
| Left Attention Network | 0.52 | 0.37 | <0.001 |
| Left Salience Network | 0.65 | 0.36 | <0.001 |
| Left Limbic Network | 0.53 | 0.42 | <0.001 |
| Left Control Network | 0.72 | 0.35 | <0.001 |
| Left Default Mode Network | 0.65 | 0.37 | <0.001 |
| Right Visual Network | 0.74 | 0.47 | <0.001 |
| Right Sensorimotor Network | 0.41 | 0.13 | <0.05 |
| Right Attention Network | 0.52 | 0.29 | <0.001 |
| Right Salience Network | 0.56 | 0.34 | <0.001 |
| Right Limbic Network | 0.32 | 0.43 | <0.05 |
| Right Control Network | 0.65 | 0.36 | <0.001 |
| Right Default Mode Network | 0.66 | 0.39 | <0.001 |
| **ADNI** | | | |
| Left Visual Network | 0.37 | 0.29 | <0.01 |
| Left Attention Network | 0.24 | 0.44 | <0.05 |
| Left Salience Network | 0.41 | 0.44 | <0.01 |
| Left Limbic Network | 0.28 | 0.28 | <0.05 |
| Left Control Network | 0.34 | 0.38 | <0.01 |
| Left Default Mode Network | 0.32 | 0.47 | <0.05 |
| Right Visual Network | 0.47 | 0.45 | <0.001 |
| Right Attention Network | 0.37 | 0.54 | <0.01 |
| Right Salience Network | 0.36 | 0.41 | <0.01 |
| Right Control Network | 0.36 | 0.38 | <0.01 |
| Right Default Mode Network | 0.37 | 0.48 | <0.01 |
